# The Functional Characterization of the 6-Phosphogluconate Dehydratase Operon in 2-Ketogluconic Acid Industrial Producing Strain *Pseudomonas plecoglossicida* JUIM01

**DOI:** 10.3390/foods13213444

**Published:** 2024-10-28

**Authors:** Wen-Jing Sun, Qian-Nan Zhang, Lu-Lu Li, Meng-Xin Qu, Xin-Yi Zan, Feng-Jie Cui, Qiang Zhou, Da-Ming Wang, Lei Sun

**Affiliations:** 1 School of Food and Biological Engineering, Jiangsu University, Zhenjiang 212013, China; juswj@163.com (W.-J.S.); zhangqiannan9912@163.com (Q.-N.Z.);; 2 Jiangxi Provincial Engineering and Technology Center for Food Additives Bio-Production, Shangrao 334221, China; 3 Key Laboratory of Elemene Class Anti-Cancer Medicines, School of Pharmacy, Hangzhou Normal University, Hangzhou 311121, China

**Keywords:** 2-ketogluconic acid (2KGA), *Pseudomona*s *plecoglossicida*, 6-phosphogluconate dehydratase (*edd*) operon, characterization

## Abstract

Genus *Pseudomonas* bacteria mainly consume glucose through the Entner–Doudoroff (ED) route due to a lack of a functional Embden–Meyerhof–Parnas (EMP) pathway. In the present study, a 6-phosphogluconate dehydratase (*edd*) operon in the ED route was well investigated to find its structural characteristics and roles in the regulation of glucose consumption and 2-ketogluconic acid (2KGA) metabolism in the industrial 2KGA-producer *P. plecoglossicida* JUIM01. The *edd* operon contained four structural genes of *edd*, *glk*, *gltR*, and *gtrS*, encoding 6-PG dehydratase Edd, glucokinase Glk, response regulatory factor GltR, and histidine kinase GtrS, respectively. A promoter region was observed in the 5′-upstream of the *edd* gene, with a transcriptional start site located 129 bp upstream of the *edd* gene and in a pseudo-palindromic sequence of 5′-TTGTN_7_ACAA-3′ specifically binding to the transcription factor HexR. The knockout of the *edd* gene showed a remarkably negative effect on cell growth and re-growth using 2KGA as a substrate, beneficial to 2KGA production, with an increase of 8%. The deletion of *glk* had no significant effect on the cell growth or glucose metabolism, while showing an adverse impact on the 2KGA production, with a decrease of 5%. The outputs of the present study would provide a theoretical basis for 2KGA-producer improvement with metabolic engineering strategies and the development and optimization of *P. plecoglossicida* as the chassis cells.

## 1. Introduction

2-Ketogluconic acid (2KGA) is a noncorrosive organic acid (pKa = 2.66) with multiple uses, like as a calcium-enriched feed additive, cement plasticizer, cement retarder, and detergent [1,2]. 2KGA is also utilized as a building block for the synthesis of heterocyclic compounds and stereoselective or regioselective chemicals. The most important application of 2KGA is in the food industry as an intermediate for the production of GRAS (generally recognized as safe) antioxidant D-isoascorbic acid (E315-free acid and E316-sodium salt) to maintain food color/flavors and block the formation of ammonium nitrite (carcinogenic) during food processing [3,4].

**Figure 1:**
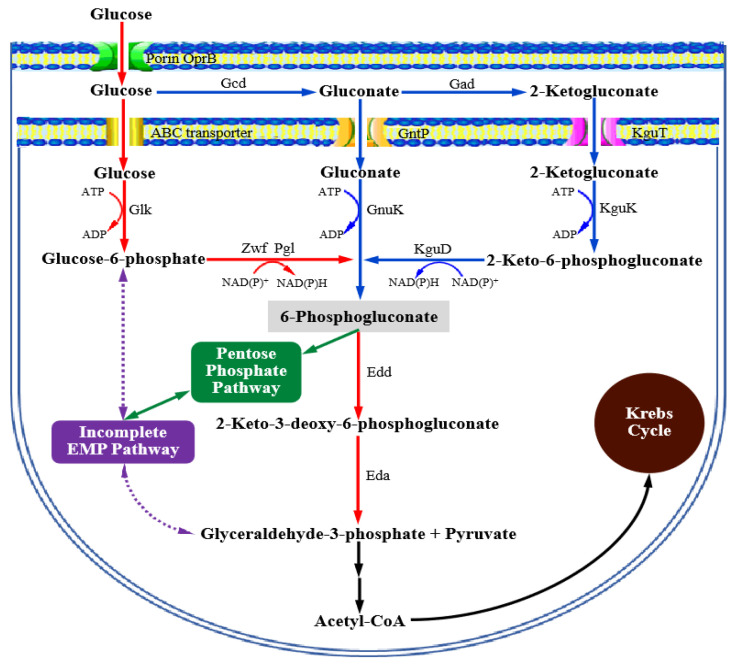
The deduced glucose metabolism in *Pseudomonas* based on the gene annotations and functional analysis.

The genus *Pseudomonas* bacteria have the glucose oxidation system located in the periplasmic space, which is mainly composed of membrane-bound pyrroloquinoline quinone (PQQ)-dependent glucose dehydrogenase (Gcd) and FAD-dependent gluconate dehydrogenase (Gad). The Gcd and Gad successively oxidize glucose to gluconic acid and gluconic acid to 2KGA in the periplasmic space [5,6,7,8]. Most interestingly, members of the genus *Pseudomonas* show significant adaptation, survival, and persistence capacities in response to environmental cues and stresses, which are beneficial for their industrial processes [9,10,11,12,13]. *Pseudomonas* bacteria have a specific glucose phosphorylation system that generates 6-phosphogluconate from glucose via three parallel pathways [14,15]. Glucose, gluconate, and 2KGA in the periplasmic space could be transported into the cell cytoplasm by the specific corresponding ATP-dependent transporters [16]. Generally, glucose enters the cell cytosol through an ABC transporter and is phosphorylated to glucose-6-phosphate by glucokinase (Glk), then further converted to 6-phosphogluconate (6-PG) via the sequential activity of glucose-6-phosphate-dehydrogenase (Zwf) and 6-phosphogluconolactonase (Pgl). The second pathway involves the transport of gluconic acid by gluconate permease (GntP), following the direct phosphorylation of gluconic acid to 6-PG mediated by gluconokinase (GnuK), and the third pathway, also called the 2KGA loop, involves the transport of 2KGA via 2-ketogluconate transporter (KguT) into cytosol, phosphorylation to 2-keto-6-phosphogluconate (2K6PG) via 2KGA kinase (KguK), and conversion into 6-PG via 2K6PG reductase (KguD). The absence of 6-phosphofructo-1-kinase (Pfk) in the genus *Pseudomonas* leads to the incomplete Embden–Meyerhof–Parnas pathway (EMP pathway). Hence, the glucose metabolism in genus *Pseudomonas* usually utilizes the ED-EMP route [14,15,16,17,18,19], which includes the Entner–Doudoroff pathway (ED pathway), pentose phosphate pathway (PP pathway), and partial EMP pathway (Figure 1). The metabolic flux analysis proved that over 90% of the consumed sugar was converted as 6-phosphogluconate in *P. putida* KT2440, which was further consumed in the ED pathway to 2-keto-3-deoxy-6-phosphogluconate (KDPG) by 6-PG dehydratase (Edd), further split by KDPG aldolase (Eda) into two trioses, pyruvate (Pyr) and glyceraldehyde-3-P (Glc-3-P), for further metabolism [16,20,21]. Less than 10% of 6-phosphogluconate entered the PP pathway, proving that the ED pathway plays the predominant role in glucose uptake in the genus *Pseudomonas* [19,21].

The transcriptional regulation of the ED pathway has been well investigated in *P. putida* and *P. aeruginosa*. The operon *edd*/*glk*/*gltR*/*gtrS* in *P. putida* contains four structure genes encoding proteins Edd, Glk, GtrS, and GltR, respectively, while the other operon *zwf/pgl/eda* had three structure genes encoding proteins Zwf, Pgl, and Eda, respectively [20,22]. Among them, GtrS and GltR form a two-component regulatory system (TCS) [23]. TCSs play crucial roles in bacterial adaptation to environments. It has been shown that many TCSs are involved in antibiotic resistance and the virulence of Gram-negative pathogens, such as *Klebsiella pneumoniae*, *Acinetobacter baumannii*, and *Pseudomonas aeruginosa* [24]. Since the Edd and Eda are the typical enzymes in the ED pathway [14], the *edd/glk/gltR/gtrS* operon and *zwf/pgl/eda* operon in the present context were nominated as the *edd* operon and *eda* operon, respectively. The pseudo-palindromic consensus sequences of 5′-TTGTN_7–8_ACAA-3′ in the promoters of the *edd* operon and the *eda* operon specifically bind the transcriptional regulator of HexR to control the transcriptional expression of the operons [18]. KDPG is the specific effector molecule of HexR, meaning that KDPG is the molecular inducer of *edd* and *eda* operons to activate the transcription of target genes by binding the sugar isomerase-like binding (SIS) domain at their C-terminal extension of HexR [25].

The *edd* operon is one of the key operons involved in glucose metabolism in the genus *Pseudomonas* participating in the transport, metabolism, and regulation of glucose [23]. However, the publications related to the *edd* operon are available in *P. aeruginosa* [18,26] and *P. putida* [22,27]. *P. plecoglossicida* JUIM01 is a biosafe 2KGA producer with high yield and has been applied in industrial production for over ten years, while its resistance (for example, tolerance to high temperature and high concentrations of 2KGA and substrate glucose) and production efficiency still need to be further improved [28]. Hence, the present study would focus on elucidating the structural characteristics, function, and expression regulation of the *edd* operon of *P. plecoglossicida* JUIM01, and analyzing the relationship between the *edd* operon and the 2KGA metabolic pathway to provide a theoretical basis for the improvement of 2KGA producers using the metabolic regulation strategies.

## 2. Materials and Methods

### 2.1. Bacterial Strains, Plasmids, Media, and Growth Conditions

Bacterial strains and plasmids used in this study are listed in Table S1. *E. coli* JM109 and DH5α were used in the present study and grown in LB medium at 37 °C for cloning and storing constructed plasmids. *P. plecoglossicida* JUIM01 is an industrial 2KGA-producing strain screened and stored in our laboratory [28,29]. *P. plecoglossicida* cells were activated on the media containing 10 g/L peptone, 5 g/L beef extract, 5 g/L NaCl, and 20 g/L agar at pH 7.0. The activated cells were inoculated into the 50 mL of seed media consisting of 20 g/L glucose, 5 g/L corn syrup powder, 2 g/L urea, 2 g/L KH_2_PO_4_, 0.5 g/L MgSO_4_·7H_2_O, and 5 g/L CaCO_3_ at pH 7.0 in the 500 mL Erlenmeyer flask and cultivated at 33 °C and 265 rpm for 20 h to harvest the seed. The 2KGA fermentation was conducted by inoculating 10% (*v*/*v*) of the seed into the 40 mL of fermentation media containing 180 g/L glucose, 10 g/L corn syrup powder, and 45 g/L CaCO_3_ in the 500 mL Erlenmeyer flask and cultivating it at 33 °C and 265 rpm.

### 2.2. Bioinformatics Analysis of the Edd Operon from P. plecoglossicida JUIM01

The plasmids used in the present study were listed in Table S2. *P. plecoglossicida* JUIM01 genome DNA was extracted using a Bacteria Genomic DNA Kit (TIANGEN, Tianjin, China). The *edd* operon gene was cloned using the extracted genome DNA as the template and the pairs of primers *edd*-P1/P2 by long and accurate polymerase chain reaction (LA-PCR). The cloned product was sequenced by Sangon Biotech Co. (Shanghai, China).

The promoter and structural genes and their positions of *edd* operon were predicted by using online ORF Finder (https://https.ncbi.nlm.nih.gov/orffinder/, accessed on 31 October 2022) and BPROM (http://www.softberry.com/, accessed on 24 April 2023). NCBI Protein BLASTn (https://www.ncbi.nlm.nih.gov/, accessed on 31 October 2022) was used to align the structural gene-encoded amino acid sequences in the *edd* operon [30]. Pfam online software (http://pfam.xfam.org/search/sequence, accessed on 16 January 2023) and Phobiusonline software (http://phobius.sbc.su.se/, accessed on 16 January 2023) were used to analyze protein domains, transmembrane structure, and predicted signal peptides [31,32].

### 2.3. Co-Transcription Assay of Structural Genes in the Edd Operon

*P. plecoglossicida* cells were collected after cultivating in seed media for 12–14 h at 32 °C and 265 rpm. The total RNA was extracted and purified using the bacteria total RNA extraction kit and RNase-free DNA removal kit (Sangon Biotech, Shanghai, China). The cDNA was prepared by reverse transcription using a FastKing RT Kit (with gDNase) (TIANGEN, Tianjin, China). The co-transcriptions of *edd*, *glk*, *gltR,* and *gtrS* were checked using the designated primers of G1/G2, G3/G4, and G5/G6 (Table S2) to clone the transgene fragments of *glk-gltR*, *edd-gltR,* and *edd-gtrS* with *P. plecoglossicida* JUIM01 genome DNA (positive), DI water (negative), and cDNA as the templates.

### 2.4. Promoter Fusion and β-Galactosidase Activity Assay

Based on the predicted sequence of the promoter region in the *edd* operon, a fragment of the *gapA-edd* complex region containing the 40 bp terminal homologous sequence of the linearized pME6522 was amplified using E1/E2 as primers, and the gene fragment was digested with restriction endonucleases *Pst* I and *Eco*R I. The seamless clone was used to ligate the purified target fragment with the linearized pME6522 to obtain the recombinant promoter probe plasmid pME6522-*edd* containing the fused sequence of the upstream fragment of *edd* and *lac*Z reporter gene in the promoter probe. The pME6522-*edd* and pME6522 were electro-transferred into *P. plecoglossicida* competent cells to generate the positive transformants of JUIM01/pME6522-*edd* and JUIM01/pME6522, respectively. The JUIM01/pME6522-*edd* and JUIM01/pME6522 strains were cultured in the LB media containing 20 g/L of glucose and 10 μg/mL of tetracycline at 32 °C and 200 r/min. The cell density (OD_600 nm_) at 12 h and 24 h were determined. The β-galactosidase activity of JUIM01/pME6522 and JUIM01/pME6522-*edd* cells at 12 h and 24 h was determined using the β-Galactosidase Enzyme Assay System with Reporter Lysis Buffer (Promega, Madison, WI, USA). The β-galactosidase activity was calculated as follows:

(1) $$\mathrm{Miller}\;\mathrm{units}=\frac{1000\;\times \;\;({\mathrm{OD}}_{420}\;-\;{\mathrm{OD}}_{550}\times 1.75)}{T\;\times \;V\;\times \;{\mathrm{OD}}_{600}}$$

Herein, T represented the reaction time (min), V represented the sample volume added in the reaction system (mL), and OD_600_ represented the absorbance of cell density at 600 nm. In comparison, OD_420_ and OD_550_ represented the absorbance at 420 nm and 550 nm after reaction, respectively.

### 2.5. 5-Rapid Amplification of cDNA Ends (5′-RACE)

The transcription start site (TSS) of the *edd* operon was determined using a 5′-RACE kit (Sangon Biotech, Shanghai, China) with the primers shown in Table S3. Using RT1/RT2 as primers, the cDNA was synthesized by reverse transcription using the total RNA of *P. plecoglossicida* JUIM01 as the template and digested by RNase H-, and a poly-C tail was added using deoxynucleotidyl transferase (TDT). The second-run PCR was conducted using cDNA and the above PCR product as the templates and NR1/adaptor and NR2/outer as the primers. The second-run PCR product was added a poly-A tail and ligated with pMD20-T to construct the plasmid pMD20-T-E. The constructed plasmid pMD20-T-E was transferred to *E. coli* JM109 to screen the positive transformants with LB plates containing 50 μg/mL ampicillin for further sequencing.

### 2.6. Construction of the Structural Gene-Deficient Strains and Corresponding Gene-Complemented Strains

The plasmid pK18*mobsacB* was used for the construction of a series of *P. plecoglossicida* JUIM01 mutants deficient with the structural genes of *edd*, *glk*, *gltR*, and *gtrS* in the *edd* operon using two-step homologous recombination [29]. Briefly, using the genomic DNA of *P. plecoglossicida* JUIM01 as a template, the upstream and downstream fragments of the structural genes of *edd*, *glk*, *gltR*, and *gtrS* were amplified using the primers of R1/R2 and R3/R4 (Tables S1 and S2), respectively. These corresponding upstream and downstream fragments of *edd*, *glk*, *gltR*, and *gtrS* were used as the templates and fused using Touchdown PCR using R1/R4 as primers to obtain the fused fragments of Δ*edd*, Δ*glk*, Δ*gltR*, and Δ*gtrS*, respectively.

Since the primers R1/R4 of Δ*glk* contained the homogenous sequence of the suicide plasmid pK18*mobsacB* [29], the plasmid pK18Δ*glk* was constructed by ligating the Δ*glk* fragment with linearized pK18*mobsacB* using the seamless cloning method. Similarly, suicide plasmids of pK18Δ*edd*, pK18Δ*gltR*, and pK18Δ*gtrS* were constructed by inserting the cloned Δ*edd*, Δ*gltR*, and Δ*gtrS* fragments into the *Bam*H I and *Hin*d III restriction sites of pK18*mobsacB*, respectively, since the primers R1/R4 of Δ*edd*, Δ*gltR*, and Δ*gtrS* fragments contained the *Bam*H I and *Hin*d III restriction sites. These suicide plasmids of pK18Δ*edd*, pK18Δ*glk*, pK18Δ*gltR*, and pK18Δ*gtrS* were electro-transformed into *P. plecoglossicida* competent cells, which resulted in the first recombination. The selected colonies were cultivated for 18 h in a non-selective LB medium and subjected to the second recombination. Subsequently, an appropriate amount of the LB culture was plated onto LB agar containing 10% (*w*/*v*) sucrose to select *edd-*, *glk-*, *gltR-*, or *gtrS-*deleted strains. The modified genotype was investigated by PCR analysis, and the positively identified strain was named JUIM01Δ*edd*, JUIM01Δ*glk*, JUIM01Δ*gltR*, and JUIM01Δ*gtrS*, respectively.

The plasmid pBBR1MCS-2 was used for the construction of *edd-*, *glk-*, *gltR-*, or *gtrS-*complemented mutants [29]. Based on the primers R5/R6 containing the *Hin*d III and *Bam*H I restriction sites (Table S2), the *edd*, *glk*, *gltR*, and *gtrS* genes were amplified with the genomic DNA of *P. plecoglossicida* JUIM01 as the template, respectively. The PCR products and pBBR1MCS-2 were digested with *Hin*d III and *Bam*H I and ligated to obtain pBBR*edd*, pBBR*glk*, pBBR*gltR*, and pBBR*gtrS*, respectively. These plasmids were electro-transformed into JUIM01Δ*edd*, JUIM01Δ*glk*, JUIM01Δ*gltR*, and JUIM01Δ*gtrS* cells, respectively, and the complemented mutants JUIM01Δ*edd-edd*, JUIM01Δ*glk-glk*, JUIM01Δ*gltR-gltR*, and JUIM01Δ*gtrS-gtrS* were finally obtained after screening on the selective plates containing 25 μg/mL of kanamycin for sequencing.

### 2.7. Analytical Methods

The cell growth during seed culture and 2KGA fermentation was represented by optical density at 650 nm (OD_650 nm_) using a Biospec-1601 spectrophotometer (Shimadzu, Japan) or the dry cell weight (DCW) computed from a curve relating OD_650 nm_ to DCW. An OD_650 nm_ of 1.0 represents 0.575 g dry cell weight per liter. Glucose concentration was determined with a Biosensor Analyzer (Shandong Academy of Sciences Institute of Biology, Jinan, China) at 25 °C. The concentration of 2KGA was determined and calculated based on glucose concentration using the Polarimetry method developed by our group [33]. The determining procedure was described briefly as follows: a sample of cultured broth was first centrifuged at 4000 r/min for 20 min, and 25 mL of resulting supernatant was mixed with 20 mL of deionized water, adjusted to pH of 1.5 by adding 1 M HCl, and then diluted to 100 mL with deionized water. The final sample solution’s optical rotation degree was determined with a WZZ-1SS Digital Automatic Polarimeter (Precision Instrument Co., Ltd., Shanghai, China). The 2KGA concentration was calculated with the following Equation (2):

Y = −0.88 X1 + 0.5275 X2(2)

X1 and X2 represented 2KGA and glucose concentrations, (g/L), respectively. Y represented optical rotation degree (°). Coefficients −0.88 and 0.5275 represented the optical rotation degree of 10.0 g/L of 2KGA and 10.0 g/L of glucose, respectively.

### 2.8. Statistical Analysis

All experiments were performed in three replicates. The data were expressed as the mean ± standard deviation (*n* = 3). The data sets were evaluated by one-way analysis of variance (ANOVA). Statistical comparisons were made based on the *P* value (α = 0.05 and 0.01).

## 3. Results and Discussion

### 3.1. Identification of Structural Genes in the Edd Operon of P. plecoglossicida JUIM01

**Figure 2:**
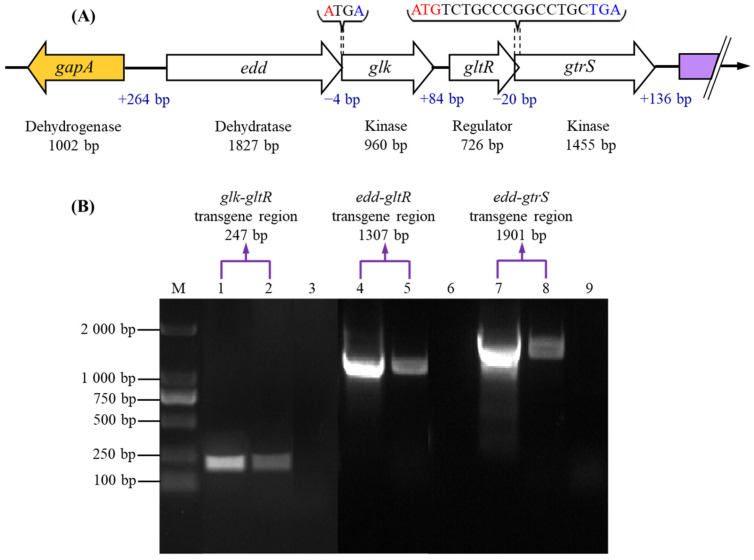
The physical map of the putative *edd* operon (**A**), and co-transcription assay of *edd*, *glk*, *gltR*, and *gtrS* in the putative *edd* operon (**B**). (Lane M, DL 2000 bp DNA marker; lane 1, using gDNA as a template (primer pair G1/G2); lane 2, using cDNA as a template (primer pair G1/G2); lane 3, using deionized water as a template (primer pair G1/G2); lane 4, using gDNA as a template (primer pair G3/G4); lane 5, using cDNA as a template (primer pair G3/G4); lane 6, using deionized water as a template (primer pair G3/G4); lane 7, using gDNA as a template (primer pair G5/G6); lane 8, using cDNA as a template (primer pair G5/G6); and lane 9, using deionized water as a template (primer pair G5/G6).

A 6591 bp *edd* operon was cloned using a pair of primers of P1 and P2 from *P. plecoglossicida* JUIM01. The *edd* operon contained five open reading frames (ORFs). ORF1 showed an opposite transcript direction with other ORFs (ORFs 2–5) (Figure 2A and Table 1). ORF1, with an entire length of 1002 bp, encoded a hydrophilic protein containing 1781 amino acid residues localized to the cytoplasm, sharing 100% homology with that of glyceraldehyde-3-phosphate dehydrogenase (catalyzing glyceraldehyde-3-phosphate to 1,3-diphosphate glycerate) of *P. putida*, proving that the ORF1 gene should be classified as the *gapA* encoding glyceraldehyde-3-phosphate dehydrogenase. The 1827 bp ORF2 located at the 265 bp upstream of *gapA* and encoded a 65.28 kDa hydrophilic protein consisting of 608 amino acid residues, sharing over 99% homology with those of Edds (catalyzing 6-PG to 2K6PG) in *P. putida* and *P. guariconensis*. ORF3 had a full length of 960 bp with 4 bp (ATGA) of overlap with that of ORF2 and encoded a 33.59 kDa hydrophobic protein localized to the cytoplasm sharing over 99% of homology with those of Glks (catalyzing glucose to glucose-6-phosphate) in *P. putida* and *Pseudomonas sp*. p1 (2021b). ORF4 was located at 86 bp downstream of ORF3 with an entire length of 726 bp and encoded a 27.27 kDa hydrophilic protein containing 241 amino acid residues localized to the cytoplasm, sharing 98% homology with a response regulatory factor (GltR) in *P. putida* and *Pseudomonas sp*. p1 (2021b). The 1455 bp ORF5 had a 20 bp overlap (ATGTCTGCCCGGCCTGCTGA) with its upstream of ORF4 and encoded a 54.15 kDa transmembrane hydrophobic protein composed of 484 amino acid residues, sharing 98% homology with a sensor histidine kinase (GtrS) in *P. putida* and *Pseudomonas sp.* p1 (2021b). ORF5 had two transmembrane helices (TMHs) at 18–40 residues and 210–228 residues, with an extra-membrane region at 210–228 residues. GtrS and GltR form a two-component regulatory system (TCS), GtrS-GltR, which regulates glucose metabolism and transport, and the expression of *toxA* encoding exotoxin A in *P. aeruginosa* [23].

### 3.2. Co-Transcription of Structural Genes in the Edd Operon

Since there are overlaps between *edd* with *glk* and *gltR* with *gtrS*, the promoters should not exist with *edd-glk* and *gltR-gtrS*. On this basis, the transgene regions between *glk* with *gltR*, *edd* with *gltR*, and *edd* with *gtrS* were amplified using G1/G2, G3/G4, and G5/G6 as primers, and cDNA and gDNA of *P. plecoglossicida* JUIM01 and ddH_2_O as templates, respectively, to verify the co-transcription of these structural genes in the *edd* operon. As shown in Figure 2B, no cloned bands were observed when using ddH_2_O as templates. The cloned *glk-gltR*, *edd-gltR*, and *edd-gtrS* showed identical 247 bp, 1307 bp, and 1901 bp bands on the agarose gel when using the cDNA and gDNA of *P*. *plecoglossicida* JUIM01 as templates, supporting the idea that *edd*, *glk*, *gltR*, and *gtrS* belonged to the structural genes of the *edd* operon and showed a co-transcription pattern, which was similar with the *edd* operon in *P. putida* [22]. Moreover, the structure of the *edd* operon may be a common characteristic among *Pseudomonas* species [34].

### 3.3. Identification of the Promoter Region of the Edd Operon

**Figure 3:**
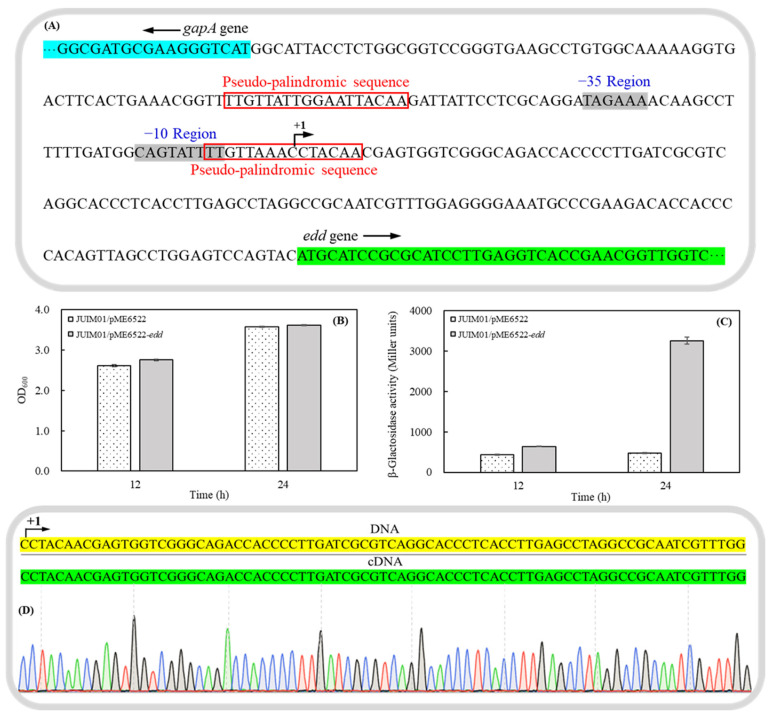
The prediction of the promoter region of the *edd* operon (**A**), a comparison of the cell growth (**B**) and β-galactosidase activities (**C**) between JUIM01/pME6522 and JUIM01/pME6522-*edd* after 12 h and 24 h cultivation, and the determined transcription start site of the *edd* operon by 5′-RACE (**D**).

The promoter region of the *edd* operon in *P. plecoglossicida* JUIM01 was predicted using the online software BPROM (https://www.softberry.com/berry.phtml?topic=bprom&group=programs&subgroup=gfindb, accessed on 24 April 2023). As shown in Figure 3A, the −10 region in *edd* operon with the sequence of CAGTATTTT was the RNA polymerase binding site, and the −35 region of TAGAAA was the recognition site of RNA polymerase σ factor. The transcriptional start site (+1) was located at 129 bp upstream of the *edd* gene and in a pseudo-palindromic sequence of TTGTN_7_ACAA, which specifically bound to the transcription factor HexR [34]. Additionally, another pseudo-palindromic sequence of TTGTN_10_ACAA was also observed in the promoter region with potential roles to regulate the transcription of *gapA*.

On the basis of the predicted promoter region, a 287 bp fragment upstream of the *edd* gene was cloned and fused with *lacZ* reporter in the promoter probe vector pME6522 to construct the pME6522-*edd* (Figure S1) and the corresponding mutants of JUIM01/pME6522 and JUIM01/pME6522-*edd*. As shown in Figure 3B,C, mutant JUIM01/pME6522 and JUIM01/pME6522-*edd* cells showed the same growth trend and amount after 12 h and 24 h cultivation, while the produced β-galactosidase activities of JUIM01/pME6522-*edd* cultivated at 12 h and 24 h were 1.6 and 6.88 times those of JUIM01/pME6522, respectively. Hence, it could be proven that a promoter region was located 5′-upstream of the *edd* gene, and *edd*, *glk*, *gltR*, and *gtrS* genes should belonged to the structural genes of the *edd* operon. The transcriptional start site was confirmed 129 bp upstream of the translation initiation site of the *edd* gene with the first base, C (Figure 3D), in consistency with the previous prediction.

### 3.4. The Roles of the Structural Genes of the Edd Operon in 2KGA Metabolism

**Figure 4:**
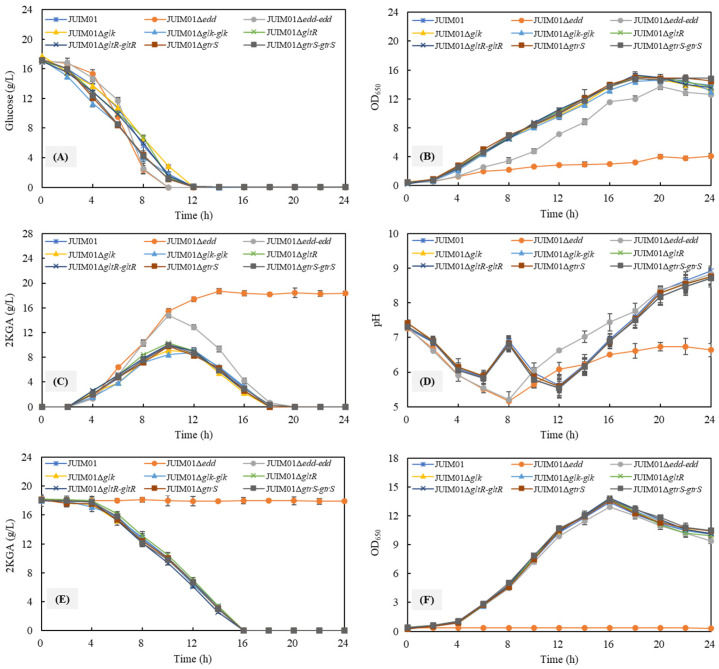
The growth and metabolism of *P. plecoglossicida* JUIM01 and its mutant strains using glucose (**A**–**D**) or 2KGA (**E**,**F**) as the sole carbon source.

The structural gene-knocked mutants of JUIM01Δ*edd*, JUIM01Δ*glk*, JUIM01Δ*gltR*, and JUIM01Δ*gtrS* (Figure S6) and their complemented strains of JUIM01Δ*edd-edd*, JUIM01*Δglk-glk*, JUIM01Δ*gltR-gltR*, and JUIM01Δ*gtrS-gtrS* (Figure S8) were constructed, respectively, to elucidate the roles of *edd*, *glk*, *gltR*, and *gtrS* genes in the 2KGA metabolism of *P. plecoglossicida* JUIM01. Figure 4A–D present the differences in the cell growth and 2KGA metabolism of the parent strain *P. plecoglossicida* JUIM01 and the structural gene-knocked and complemented strains when using 20 g/L of glucose as the sole carbon source. The knockout of *glk*, *gltR*, or *gtrS* had no significant changes in glucose utilization, cell growth, 2KGA synthesis and consumption, or pH levels in broth. Interestingly, the deletion of *edd* and the complementation of *edd* to the JUIM01Δ*edd* strain benefited the glucose consumption rates of over 1.8 g/L/h, which increased by 20% compared to the parent strain. The *edd*-knocked strain JUIM01Δ*edd* showed a high 2KGA production efficiency of the produced 2KGA concentration of 18.2 g/L and could not use the produced 2KGA as the sole carbon source (Figure 4E,F). The deletion of *edd* resulted in a remarkable reduction in cell concentration and growth rate with the maximum cell concentration of 2.35 g/L (OD_650 nm_ = 4.08) compared to those of the parent strain with the maximum cell concentration of 8.56 g/L (OD_650 nm_ = 14.89). Corresponding to an efficient 2KGA production performance, the pH of JUIM01Δ*edd* broth also showed a significant difference from that of JUIM01.

Generally, 6-PG dehydratase encoded (Edd) by the *edd* gene is a key enzyme in the ED pathway. Not only in glucose metabolism, Edd also plays an important role in glycerol utilization and the biofilm phenotypes and virulence of *P. aeruginosa*, and it contributes to root colonization and the induction of the systemic resistance of *Pseudomonas chlororaphis* [19,35]. The *edd*-deletion in *P. putida* resulted in a block of glucose utilization via the ED pathway and led to the readjustment of the metabolic pathway to produce fructose 6-phosphate and a decrease in the cell growth rate [36]. Actually, the decreased cell concentration and growth rate originated from the inability of the genus *Pseudomonas* to utilize the produced 2KGA as the carbon source for 2nd-run growth. del Castillo et al. had revealed that the knockout of *glk* significantly decreased the growth rate of *P. putida* KT2440 by 26.7% compared to the parent strain [20]. However, in the present study, the deletion of *glk* had no significant effect on the cell growth of *P. plecoglossicida* JUIM01, indicating that the glucose phosphorylation catalyzed by glucokinase (Glk) was dispensable to the cell growth. The existed references have proven that the 6-PG produced from the direct phosphorylation of glucose accounts for 14–17% of the synthesized 6-PG in the glucose metabolic pathway [16], indicating the significant importance of the phosphorylation of gluconic acid and 2KGA for *P. plecoglossicida* growth.

### 3.5. Production Performance of P. plecoglossicida JUIM01 and Its Mutant Strains

The 2KGA production performances of *P. plecoglossicida* JUIM01; gene-knocked mutants of JUIM01Δ*edd*, JUIM01Δ*glk*, JUIM01Δ*gltR*, and JUIM01Δ*gtrS*; and their complemented strains of JUIM01Δ*edd-edd*, JUIM01Δ*glk-glk*, JUIM01Δ*gltR-gltR*, and JUIM01Δ*gtrS-gtrS* were compared with a high C/N ratio media containing 180 g/L glucose (Table 2). As summarized in Table 2, JUIM01 and mutants had no significant differences in glucose consumption or cell growth, while there were differences in 2KGA production. The JUIM01Δ*edd* strain had the highest 2KGA concentration of 166.0 g/L and yield of 1.0 g/g (over 95.0% of the theoretical yield), increased by 8.38% compared to JUIM01, the underlying mechanism of which remains to be elucidated. Interestingly, unlike the significant difference in cell growth during seed culture, the *edd* knockout only resulted in a 3.5% decrease in the maximum cell concentration of JUIM01 during 2KGA fermentation. This probably was because of the higher initial glucose and C/N ratio in the fermentation medium, but it still needs further investigation. JUIM01Δ*glk* showed a 5.2% decrease in the 2KGA concentration of 145.2 g/L and yield of 0.9 g/g (83.2% of the theoretical yield). In addition, the gene complementation could convert the gene-knockout mutants’ 2KGA production performance back into a similar level of the wild-type strain JUIM01.

## 4. Conclusions

The *edd* operon of the industrial 2KGA-producer *P. plecoglossicida* JUIM01 was extensively characterized using reverse transcription PCR, promoter fusion, and 5′-RACE and analyzing the roles of each structural genes in the synthesis and metabolism of 2KGA for the first time. The obtained results proved that the *edd* operon of *P. plecoglossicida* JUIM01 contained four structural genes of *edd*, *glk*, *gltR*, and *gtrS*, encoding 6-PG dehydratase, glucokinase, response regulatory factor GltR, and histidine kinase GtrS, respectively. GtrS and GltR formed a two-component regulatory system of GtrS-GltR. A promoter region was observed in the 5′ upstream of the *edd* gene, with a transcriptional start site located 129 bp upstream of the *edd* gene and in a pseudo-palindromic sequence of TTGTN_7_ACAA, which specifically bound to the transcription factor HexR. The deletion of *gltR* or *gtrS* had no significant effect on the cell growth, glucose consumption, or 2KGA metabolism of *P. plecoglossicida* JUIM01. The knockout of *edd* showed a remarkably negative effect on cell growth and re-growth using 2KGA as a substrate. However, it benefited 2KGA production, with an increase of over 8% compared to JUIM01. The deletion of *glk* had no significant effect on cell growth or glucose metabolism while showing an adverse impact on 2KGA production, with a decrease of 5%. The outputs of the present study would provide a theoretical basis for 2KGA-producer improvement with metabolic engineering strategies and the development and optimization of *P. plecoglossicida* as the chassis cells.

## Figures and Tables

**Table 1:** The predicted proteins encoded by ORFs 2–5 and their similarities to other proteins in *Pseudomonas*.

ORF	Molecular Weight of the Product (No. of Amino Acid Residues)	Proposed Functionof Gene Product	Similar Gene Product (% Identity)
ORF2 (*edd*)	65.28 kDa (608)	6-Phosphogluconate dehydratase	*P. putida* and *P. guariconensis* 6-phosphogluconate dehydratase (>99%)
ORF3 (*glk*)	33.59 kDa (319)	Glucose kinase	*P. putida* and *Pseudomonas* sp. p1 (2021b) glucose kinase (>99%)
ORF4 (*gltR*)	27.27 kDa (241)	Response regulatory factor GltR	*P. putida* and *Pseudomonas* sp. p1 (2021b) response regulatory factor GltR (>98%)
ORF5 (*gtrS*)	54.15 kDa (484)	Histidine kinase GtrS	*P. putida* and *Pseudomonas* sp. p1 (2021b) histidine kinase GtrS (>98%)

**Table 2:** Comparison of 2KGA production performance between *P. plecoglossicida* JUIM01 and its mutant strains.

Strains	Initial Glucose (g/L)	Residual Glucose (g/L)	Maximum Cell Concentration (OD_650 nm_)	2KGA (g/L)	2KGA Yield (g/100 g)	Fermentation Period (h)	2KGA Productivity (g/L·h)
JUIM01	162.00	0.03 ± 0.00	9.63 ± 0.03	153.1 ± 3.8	94.5 ± 2.4	72.0	2.13 ± 0.05
JUIM01Δ*edd*	162.00	0.00 ± 0.00	9.29 ± 0.05	166.0 ± 6.4	102.4 ± 4.0	72.0	2.30 ± 0.09
JUIM01Δ*edd-edd*	162.00	0.01 ± 0.00	9.54 ± 0.04	155.9 ± 6.1	96.3 ± 3.7	72.0	2.17 ± 0.08
JUIM01Δ*glk*	162.00	0.02 ± 0.00	9.86 ± 0.02	145.2 ± 5.2	89.7 ± 3.2	72.0	2.02 ± 0.07
JUIM01Δ*glk-glk*	162.00	0.01 ± 0.00	9.71 ± 0.01	152.0 ± 4.6	93.9 ± 2.9	72.0	2.11 ± 0.06
JUIM01Δ*gltR*	162.00	0.00 ± 0.00	9.36 ± 0.04	153.6 ± 3.9	94.8 ± 2.4	72.0	2.13 ± 0.05
JUIM01Δ*gltR-gltR*	162.00	0.01 ± 0.00	9.51 ± 0.02	153.0 ± 3.0	94.4 ± 1.8	72.0	2.12 ± 0.04
JUIM01Δ*gtrS*	162.00	0.00 ± 0.00	9.74 ± 0.03	153.4 ± 1.9	94.7 ± 1.2	72.0	2.13 ± 0.03
JUIM01Δ*gtrS-gtrS*	162.00	0.03 ± 0.00	9.59 ± 0.03	152.0 ± 6.9	93.8 ± 4.2	72.0	2.11 ± 0.10

## Data Availability

The original contributions presented in the study are included in the article/Supplementary Material, further inquiries can be directed to the corresponding authors.

## Supplementary Materials

The following supporting information can be downloaded at https://www.mdpi.com/article/10.3390/foods13213444/s1; Table S1: Bacterial strains and plasmids used in this study; Table S2: Primers used in this study; Table S3: Primers used in 5′-rapid amplification of cDNA ends; Figure S1: The recombinant plasmid pME6522-*edd* was verified through the digestion of the gene fragment with *Pst* I and *Eco*R I. Lane M, 15,000 bp molecular weight marker; lane 1, the double enzyme digestion products of the recombinant plasmid pME6522-*edd*; Figure S2: Obtaining the incomplete *edd* gene fragment with the upstream and downstream of the *edd* gene. Lane M, 2000 bp molecular weight marker; lane 1, the upstream fragment of the *edd* gene; lane 2, the downstream fragment of the *edd* gene; lane 3, using *edd*-R1/*edd*-R4 as primers, an incomplete *edd* fragment with the upstream and downstream of the *edd* gene obtained through the fusion of the upstream and downstream fragments of the *edd* gene. Figure S3: Obtaining the incomplete *glk* gene fragment with the upstream and downstream of the *glk* gene. Lane M, 2000 bp molecular weight marker; lane 1, the upstream fragment of the *glk* gene; lane 2, the downstream fragment of the *glk* gene; lane 3, using *glk*-R1/*glk*-R4 as primers, an incomplete *glk* fragment with the upstream and downstream of the *glk* gene obtained through the fusion of the upstream and downstream fragments of the *glk* gene. Figure S4: Obtaining the incomplete *gltR* gene fragment with the upstream and downstream of the *gltR* gene. Lane M, 2000 bp molecular weight marker; lane 1, the upstream fragment of the *gltR* gene; lane 2, the downstream fragment of the *gltR* gene; lane 3, using *gltR*-R1/gltR-R4 as primers, an incomplete *gltR* fragment with the upstream and downstream of the *gltR* gene obtained through the fusion of the upstream and downstream fragments of the *gltR* gene. Figure S5: Obtaining the incomplete *gtrS* gene fragment with the upstream and downstream of the *gtrS* gene. Lane M, 2000 bp molecular weight marker; lane 1, the upstream fragment of the *gtrS* gene; lane 2, the downstream fragment of the *gtrS* gene; lane 3, using *gtrS*-R1/*gtrS-*R4 as primers, an incomplete *gtrS* fragment with the upstream and downstream of the *gtrS* gene obtained through the fusion of the upstream and downstream fragments of the *gtrS* gene. Figure S6: The colony PCR verification of the gene-deletion strains. Lane M, 5000 bp molecular weight marker; lane 1, a PCR fragment with *edd*-R1/*edd*-R4 as primers and the wild-type strain JUIM01 as the template; lane 2, *edd-*knockout strain JUIM01Δ*edd*; lane 3, a PCR fragment with *glk*-R1/*glk-*R4 as primers and the wild-type strain JUIM01 as the template; lane 4, *glk*-knockout strain JUIM01Δ*glk*; lane 5, a PCR fragment with *gltR*-R1/*gltR*-R4 as primers and the wild-type strain JUIM01 as the template; lane 6, *gltR-*knockout strain JUIM01Δ*gltR*; lane 7, a PCR fragment with *gtrS*-R1/*gtrS*-R4 as primers and the wild-type strain JUIM01 as the template; lane 8, *gtrS*-knockout strain JUIM01Δ*gtrS*. Figure S7: The identification of the recombinant plasmids by single and double restriction endonuclease digestion. Lane M, 15,000 bp molecular weight marker; lane 1, the recombinant plasmid pBBR*edd* was digested by *Bam*H I; lane 2, the recombinant plasmid pBBR*edd* was digested by *Bam*H I and *Hin*d III; lane 3, the recombinant plasmid pBBR*glk* was digested by *Bam*H I; lane 4, the recombinant plasmid pBBR*glk* was digested by *Bam*H I and *Hin*d III; lane 5, the recombinant plasmid pBBR*gltR* was digested by *Bam*H I; lane 6, the recombinant plasmid pBBR*gltR* was digested by *Bam*H I and *Hin*d III; lane 7, the recombinant plasmid pBBR*gtrS* was digested by *Bam*H I; lane 8, recombinant plasmid pBBR*gtrS* was digested by *Bam*H I and *Hin*d III. Figure S8: The colony PCR verification of the gene-complemented strains. Lane M, 2000 bp molecular weight marker; lane 1, a PCR fragment with *edd-*1/*edd-*2 as primers and the wild-type strain JUIM01 as the template; lane 2, the *edd*-complemented strain JUIM01Δ*edd-edd*; lane 3, a PCR fragment with *glk*-1/*glk*-2 as primers and the wild-type strain JUIM01 as the template; lane 4, the *glk*-complemented strain JUIM01Δ*glk-glk*; lane 5, a PCR fragment with *gltR*-1/*gltR-*2 as primers and the wild-type strain JUIM01 as the template; lane 6, the *gltR* complemented strain JUIM01Δ*gltR*-*gltR*; lane 7, a PCR fragment with *gtrS*-1/*gtrS*-2 as primers and the wild-type strain JUIM01 as the template; lane 8, the *gtrS* complemented strain JUIM01Δ*gtrS-gtrS*.
