# Supplementary material for: The Functional Characterization of the 6-Phosphogluconate Dehydratase Operon in 2-Ketogluconic Acid Industrial Producing Strain Pseudomonas plecoglossicida JUIM01
Source: Foods. 2024 Oct 28;13(21):3444. doi: 10.3390/foods13213444 (PMC11544825; doi:10.3390/foods13213444)
Supplement: Supplementary file 1 [file foods-13-03444-s001.zip › foods-3264147-supplementary.pdf]

Supplementary materials

The Functional Characterization of the 6-Phosphogluconate Dehydratase Operon in 2-Ketogluconic Acid Industrial Producing Strain *Pseudomonas plecoglossicida* JUIM01

Table S1. Bacterial strains and plasmids used in this study

Strain or plasmid	Description	Source
Strains		
Escherichia coli JM109	General cloning strain	TaKaRa
Escherichia coli DH5α	Cloning host	TaKaRa
P. plecoglossicida JUIM01	A 2-ketogluconate high producing strain	Our lab
JUIM01Δ*edd*	*edd* knockout mutant of JUIM01	This work
JUIM01Δ*glk*	*glk* knockout mutant of JUIM01	This work
JUIM01Δ*gltR*	*gltR* knockout mutant of JUIM01	This work
JUIM01Δ*gtrS*	*gtrS* knockout mutant of JUIM01	This work
JUIM01Δ*edd-edd*	*edd* complemented mutant of JUIM01Δ*edd*	This work
JUIM01Δ*glk-glk*	*glk* complemented mutant of JUIM01Δ*glk*	This work
JUIM01Δ*gltR-gltR*	*gltR* complemented mutant of JUIM01Δ*gltR*	This work
JUIM01Δ*gtrS-gtrS*	*gtrS* complemented mutant of JUIM01Δ*gtrS*	This work
Plasmids		
pMD20-T	SP6 Promoter, ColE1 ori, Amp^r	TaKaRa
pMD20-T-E	pMD20-T containing nested PCR products	This work
pK18*mobsacB*	Suicide vector, Kan^R	Our lab
pK18Δ*edd*	pK18*mobsacB* containing incomplete *edd* sequence of JUIM01, Kan^R	This work
pK18Δ*glk*	pK18*mobsacB* containing incomplete *glk* sequence of JUIM01, Kan^R	This work
pK18Δ*gltR*	pK18*mobsacB* containing incomplete *gltR* sequence of JUIM01, Kan^R	This work
pK18Δ*gtrS*	pK18*mobsacB* containing incomplete *gtrS* sequence of JUIM01, Kan^R	This work
pBBR1MCS-2	*E. coli-Pseudomonas* shuttle vector, Kan^R	Our lab
pBBR*edd*	pBBR1MCS-2 expressing *edd* of JUIM01, Kan^R	This work
pBBR*glk*	pBBR1MCS-2 expressing *glk* of JUIM01, Kan^R	This work
pBBR*gltR*	pBBR1MCS-2 expressing *gltR* of JUIM01, Kan^R	This work
pBBR*gtrS*	pBBR1MCS-2 expressing *gtrS* of JUIM01, Kan^R	This work
pME6522	*E. coli-Pseudomonas* shuttle vector, Tc^r	Our lab
pME6522-*edd*	pME6522 containing 287 bp PCR fragment of *edd* upstream region	This work

Table S2. Primers used in this study

Primers	Sequence (5′→3′)	Description (restriction sites)
P1	GTGAGGAAGACGGTATCGAAGCGGGACT	To amplify the operon fragment consisting of the *edd*、*glk*、*gltR* and *gtrS* gene
P2	GCGAGCAGGTAACGAAGGGATGCACGAA	
edd-R1	CGCGGATCCCTGTGGTAGACGTCGGTGAGCAC	To amplify the upstream homologous fragment of the *edd* gene (*Bam*H I)
edd-R2	TCCAATGCCGAGGTGAAGGCGAATCAGCTCCAGGTA	
edd-R3	TACCTGGAGCTGATTCGCCTTCACCTCGGCATTGGA	To amplify the downstream homologous fragment of the *edd* gene (*Hin*d III)
edd-R4	CCCAAGCTTTCCCAGCAACCCGGAAAACT	
edd-R5	CCCAAGCTTATGCATCCGCGCATCCTT	To amplify the *edd* gene (*Hin*d III)
edd-R6	CGGGATCCTCATTTGAGCTGCTCCAAT	To amplify the *edd* gene (*Bam*H I)
glk-R1	CTTTCCTGCGTTATCCCTGCCGACCCTGTCCCAC	To amplify the upstream homologous fragment of the *glk* gene
glk-R2	GATCCAACGCCTGCTGCACTCCGATGTCGCCAACCA	
glk-R3	TGGTTGGCGACATCGGAGTGCAGCAGGCGTTGGATC	To amplify the downstream homologous fragment of the *glk* gene
glk-R4	TGCGTTTTCCCTTGTCGCGTATCAGCCGAGGCG	
glk-R5	CCCAAGCTTATGAAGGCCTTGCTGGTTGGCGAC	To amplify the *glk* gene (*Hin*d III)
glk-R6	CGCGGATCCTCAATGATCCAACGCCTG	To amplify the *glk* gene (*Bam*H I)
gltR-R1	CGCGGATCCCTTCACCAACAACCACTG	To amplify the upstream homologous fragment of the *gltR* gene (*Bam*H I)
gltR-R2	GTCGAGAAACAGCTTGAGGTAGGTCTGCAACAGTTC	
gltR-R3	GAACTGTTGCAGACCTACCTCAAGCTGTTTCTCGAC	To amplify the downstream homologous fragment of the *gltR* gene (*Hind* III)
gltR-R4	CCCAAGCTTCAGCAACAGCAATGCGGT	
gltR-R5	CCCAAGCTTGTGAGCTCCACCGGCAAATCGAT	To amplify the *gltR* gene (*Hin*d III)
gltR-R6	CGCGGATCCGCAGGCCGGGCAGACATGG	To amplify the *gltR* gene (*Bam*H I)
gtrS-R1	CGCGGATCCAACGTGAGCTCCACCGGCAAATC	To amplify the upstream homologous fragment of the *gtrS* gene (*Bam*H I)
gtrS-R2	CTCAAGGTCACCCGCAAGAACAGCACCACCAGCAGC	
gtrS-R3	GCTGCTGGTGGTGCTGTTCTTGCGGGTGACCTTGAG	To amplify the downstream homologous fragment of the *gtrS* gene (*Hin*d III)
gtrS-R4	CCCAAGCTTTTCTCGAACACAGTGCTGTCCT	
gtrS-R5	CCCAAGCTTATGTCTGCCCGGCCTGCTGAGC	To amplify the *gtrS* gene (*Hin*d III)
gtrS-R6	CGCGGATCCCTCGGCGCTGCGCGGC	To amplify the *gtrS* gene (*Bam*H I)
E1	ATGGCAAAAGCTTCGAATTCCTCGCAGGATAGAAAACAAGCC	To amplify the upstream fragment of the *edd* gene contains the terminal homologous sequence of the 40 bp linearized plasmid pME6522
E2	ATCCGCTCACAATTCTGCAGAGGTAGCGTTCACGGGTGG	
G1	CTGCATGAGCGGGTATTTCG	To amplify the sequence between *glk* and *gltR*
G2	TTCGCGGATTTCCTGGTC	
G3	GATGGTGATATCGTGCGGGTCG	To amplify the sequence between *edd* and *gltR*
G4	TTCGCGGATTTCCTGGTC	
G5	TTCACCTCGGCATTGGAGCAGC	To amplify the sequence between *edd* and *gtrS*
G6	GAGACCCAGATCAGGCTCGACAGC	

Table S3. Primers used in 5′-rapid amplification of cDNA ends

Primers	Sequence (5′→3′)
Adaptor	GCTGTCAACGATACGCTACGTAACGGCATGACAGTGGGIIGGGIIGGGIIG
Outer	GCTGTCAACGATACGCTACGTAAC
Inner	GCTACGTAACGGCATGACAGTG
RT1	GGCTAACTGTGGGGTGGTGTCT (f)
RT2	GGCATTTCCCCTCCAAACG (r)
NR1	CCTAGGCTCAAGGTGAGGGTG (f)
NR2	TGCCCGACCACTCGTTGTAG (r)

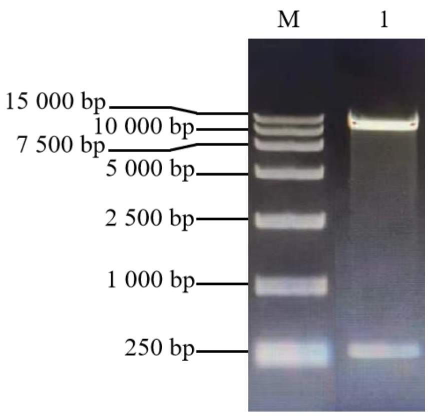

Figure S1. The recombinant plasmid pME6522-*edd* was verified through the digestion of the gene fragment with *Pst* I and *Eco*R I. Lane M, 15000 bp molecular weight marker; lane 1, the double enzyme digestion products of the recombinant plasmid pME6522-*edd*.

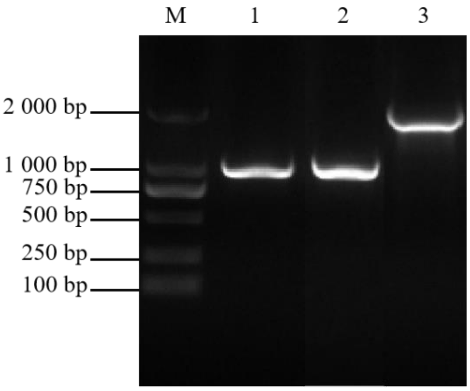

Figure S2. Obtaining the incomplete *edd* gene fragment with the upstream and downstream of the *edd* gene. Lane M, 2000 bp molecular weight marker; lane 1, the upstream fragment of the *edd* gene; lane 2, the downstream fragment of the *edd* gene; lane 3, using *edd*-R1/*edd*-R4 as primers, an incomplete *edd* fragment with the upstream and downstream of the *edd* gene obtained through the fusion of the upstream and downstream fragments of the *edd* gene.

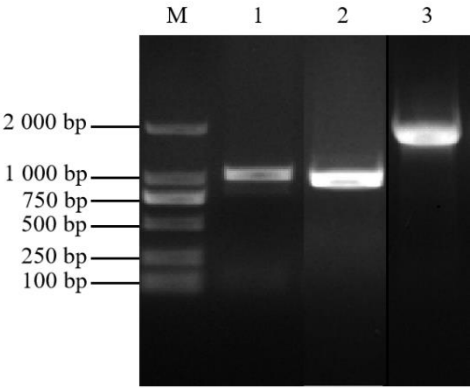

Figure S3. Obtaining the incomplete *glk* gene fragment with the upstream and downstream of the *glk* gene. Lane M, 2000 bp molecular weight marker; lane 1, the upstream fragment of the *glk* gene;

lane 2, the downstream fragment of the *glk* gene; lane 3, using *glk*-R1/*glk*-R4 as primers, an incomplete *glk* fragment with the upstream and downstream of the *glk* gene obtained through the fusion of the upstream and downstream fragments of the *glk* gene.

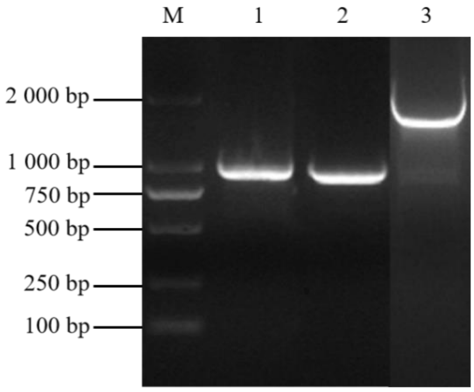

Figure S4. Obtaining the incomplete *gltR* gene fragment with the upstream and downstream of the *gltR* gene. Lane M, 2000 bp molecular weight marker; lane 1, the upstream fragment of the *gltR* gene; lane 2, the downstream fragment of the *gltR* gene; lane 3, using *gltR*-R1/*gltR*-R4 as primers, an incomplete *gltR* fragment with the upstream and downstream of the *gltR* gene obtained through the fusion of the upstream and downstream fragments of the *gltR* gene.

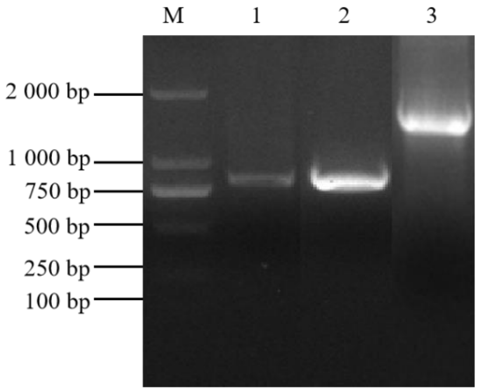

Figure S5. Obtaining the incomplete *gtrS* gene fragment with the upstream and downstream of the *gtrS* gene. Lane M, 2000 bp molecular weight marker; lane 1, the upstream fragment of the *gtrS* gene; lane 2, the downstream fragment of the *gtrS* gene; lane 3, using *gtrS*-R1/*gtrS*-R4 as primers, an incomplete *gtrS* fragment with the upstream and downstream of the *gtrS* gene obtained through the fusion of the upstream and downstream fragments of the *gtrS* gene.

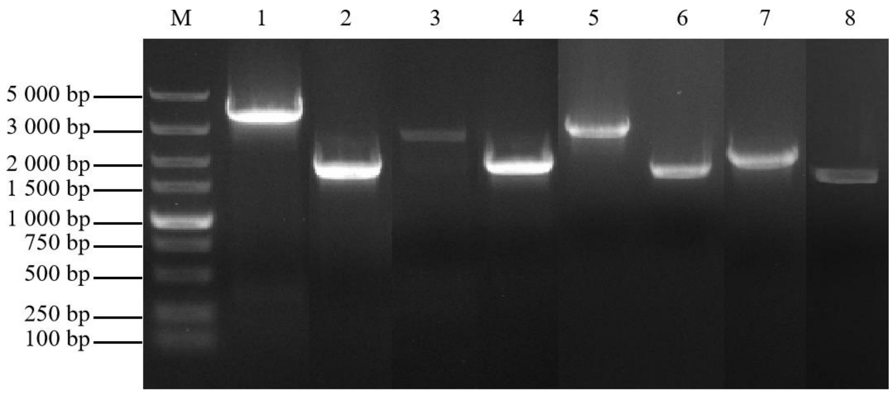

Figure S6. The colony PCR verification of the gene-deletion strains. Lane M, 5000 bp molecular weight marker; lane 1, a PCR fragment with *edd*-R1/*edd*-R4 as primers and the wild-type strain JUIM01 as the template; lane 2, the *edd*-knockout strain JUIM01Δ*edd*; lane 3, a PCR fragment with *glk*-R1/*glk*-R4 as primers and the wild-type strain JUIM01 as the template; lane 4, the *glk*-knockout strain JUIM01Δ*glk*; lane 5, a PCR fragment with *gltR*-R1/*gltR*-R4 as primers and the wild-type strain JUIM01 as the template; lane 6, the *gltR*-knockout strain JUIM01Δ*gltR*; lane 7, a PCR fragment with *gtrS*-R1/*gtrS*-R4 as primers and the wild-type strain JUIM01 as the template; lane 8, the *gtrS*-knockout strain JUIM01Δ*gtrS*.

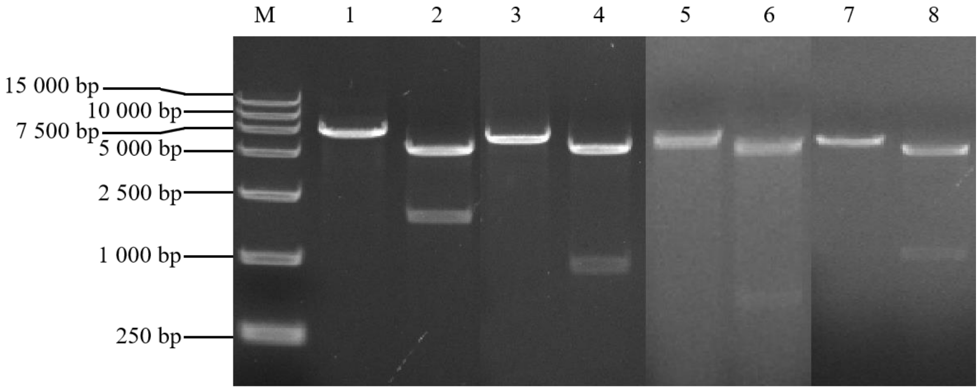

Figure S7. The identification of the recombinant plasmids by single and double restriction endonuclease digestion. Lane M, 15000 bp molecular weight marker; lane 1, the recombinant plasmid pBBR*edd* was digested by *Bam*H I; lane 2, the recombinant plasmid pBBR*edd* was digested by *Bam*H I and *Hin*d III; lane 3, the recombinant plasmid pBBR*glk* was digested by *Bam*H I; lane 4, the recombinant plasmid pBBR*glk* was digested by *Bam*H I and *Hin*d III; lane 5, the recombinant plasmid pBBR*gltR* was digested by *Bam*H I; lane 6, the recombinant plasmid pBBR*gltR* was digested by *Bam*H I and *Hin*d III; lane 7, the recombinant plasmid pBBR*gtrS* was digested by *Bam*H I; lane 8, the recombinant plasmid pBBR*gtrS* was digested by *Bam*H I and *Hin*d III.

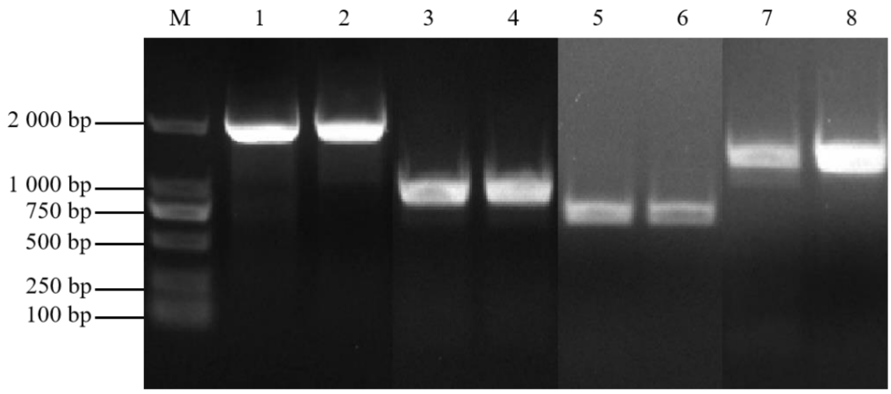

Figure S8. The colony PCR verification of the gene-complemented strains. Lane M, 2000 bp molecular weight marker; lane 1, a PCR fragment with *edd*-1/*edd*-2 as primers and the wild-type strain JUIM01 as the template; lane 2, the *edd*-complemented strain JUIM01Δ*edd*-*edd*; lane 3, a PCR fragment with *glk*-1/*glk*-2 as primers and the wild-type strain JUIM01 as the template; lane 4, the *glk*-complemented strain JUIM01Δ*glk*-*glk*; lane 5, a PCR fragment with *gltR*-1/*gltR*-2 as primers and the wild-type strain JUIM01 as the template; lane 6, the *gltR*-complemented strain JUIM01Δ*gltR*-*gltR*; lane 7, a PCR fragment with *gtrS*-1/*gtrS*-2 as primers and the wild-type strain JUIM01 as the template; lane 8, the *gtrS* complemented strain JUIM01Δ*gtrS*-*gtrS*.
